# Flowering phenophases influence the antibacterial and anti-biofilm effects of *Thymus vulgaris* L. essential oil

**DOI:** 10.1186/s12906-023-03966-1

**Published:** 2023-05-24

**Authors:** Csongor Bakó, Viktória Lilla Balázs, Erika Kerekes, Béla Kocsis, Dávid U. Nagy, Péter Szabó, Giuseppe Micalizzi, Luigi Mondello, Judit Krisch, Dóra Pethő, Györgyi Horváth

**Affiliations:** 1 Department of Pharmacognosy, Faculty of Pharmacy, University of Pécs, Pécs, H-7624 Hungary; 2 Department of Microbiology, Faculty of Science and Informatics, University of Szeged, Szeged, H-6726 Hungary; 3 Department of Medical Microbiology and Immunology, Medical School, University of Pécs, Pécs, H-7624 Hungary; 4 Institute of Geobotany and Plant Ecology, Martin-Luther University, D-06108 Halle, Germany; 5 Institute of Geography and Earth Sciences, Faculty of Sciences, University of Pécs, Pécs, H-7624 Hungary; 6 Department of Chemical, Biological, Pharmaceutical and Environmental Sciences, University of Messina, Messina, 98168 Italy; 7 Chromaleont s.r.l., c/o Department of Chemical, Biological, Pharmaceutical and Environmental Sciences, University of Messina, Messina, 98168 Italy; 8 Unit of Food Science and Nutrition, Department of Medicine, University Campus Bio-Medico of Rome, Rome, 00128 Italy; 9 Department of Food Engineering, Faculty of Engineering, University of Szeged, Szeged, H-6724 Hungary; 10 Department of MOL Hydrocarbon and Coal Processing, University of Pannonia, Veszprém, H-8200 Hungary

**Keywords:** *Thymus vulgaris*, Thyme essential oil, Phenophase, TLC-DB, Anti-biofilm effect, SEM, Thymol

## Abstract

**Background:**

Essential oils are becoming increasingly popular in medicinal applications because of their antimicrobial effect. *Thymus vulgaris* L. (Lamiaceae) is a well-known and widely cultivated medicinal plant, which is used as a remedy for cold, cough and gastrointestinal symptoms. Essential oil content of thyme is responsible for its antimicrobial activity, however, it has been reported that the chemical composition of essential oils influences its biological activity. In order to explore flowering phenophases influence on the chemical composition of thyme essential oil and its antibacterial and anti-biofilm activity, plant materials were collected at the beginning of flowering, in full bloom and at the end of flowering periods in 2019.

**Methods:**

Essential oils from fresh and dried plant materials were distilled and analyzed with gas chromatography-mass spectrometry (GC-MS) and gas chromatography-flame ionization detection (GC-FID). The antibacterial activity was performed by broth microdilution and thin layer chromatography-direct bioautography (TLC-DB) assays and the anti-biofilm effect by crystal violet assay, respectively. Scanning electron microscopy was applied to illustrate the cellular changes of bacterial cells after essential oil treatment.

**Results:**

Thymol (52.33–62.46%) was the main component in the thyme essential oils. Thyme oil distilled from fresh plant material and collected at the beginning of flowering period exerted the highest antibacterial and anti-biofilm activity against *Haemophilus influenzae*, *H. parainfluenzae* and *Pseudomonas aeruginosa*.

**Conclusion:**

The different flowering periods of *Thymus vulgaris* influence the antibacterial and anti-biofilm activity of its essential oils, therefore, the collection time has to be taken into consideration and not only the full bloom, but the beginning of flowering period may provide biological active thyme essential oil.

## Background

The growing problem of antibiotic resistance poses a continuing challenge to healthcare practitioners. The failure of therapies may due to the biofilm-forming ability of several pathogens (e.g. *Pseudomonas aeruginosa, Staphylococcus epidermidis*, methicillin-resistant *Staphylococcus aureus*, (MRSA), *Acinetobacter baumannii*, *Haemophilus* spp.) enhancing their antimicrobial resilience, host defense mechanisms, and stress resistance [1–4]. Three of the bacteria listed were selected for our experiments. *P. aeruginosa* was chosen because the prevalence of antibacterial resistant infections is steadily increasing, especially in patients with cystic fibrosis [5]. *Haemophilus* spp. were chosen because there is less literature available compared to other bacterial strains.

These bacteria start to form an extracellular matrix consists of extracellular polysaccharides, nucleic acids, and proteins which could easily accumulate on animal or human tissues, and biomaterials (e.g. contact lenses, implants, catheters, and shunts) as well [6, 7]. Thus, biofilm-forming ability plays a significant role in various diseases (e.g. cystic fibrosis, dental caries, tonsillitis, otitis media), therefore, those researches which are focusing on their eradication receiving more attention nowadays [8–11].

Application of essential oils (EOs) as natural antimicrobial agents became widespread in the food industry, pharmaceutical, and medicinal products over the past decades [12, 13]. The composition of EOs can be influenced by several factors: environmental factors, vegetation period, collection technique, distillation procedure, chemotype, and collected herbal parts as well [14–18]. Therefore, different factors may alter the quantity of the main compounds, besides it also leads to modified biological activity [19, 20]. Thus, for producing the best yield, and quality with a proper antibacterial effect, it is necessary to clarify the connection between the environmental conditions and microbiological potential as well. Based on this, the accurate determination of the chemical profile of the EOs is unquestionable [21, 22].

The antibacterial [23, 24], and antioxidant capacity [25] of *Thymus* species were described by several publications before as well as their anti-inflammatory potential [26]. *Thymus vulgaris* L. is a Mediterranean aromatic plant, which belongs to the Lamiaceae family. Among the six chemotypes, the thymol-dominated one is the most widespread in the natural habitats [18]. According to previous reports, the antimicrobial activity is probably influenced by the main compounds (thymol and carvacrol), indicated programmed cell death mechanism [27]. Besides the main compounds, other minor compounds (*α*- and *γ*-terpinene) were identified as free radical scavengers or lipid-peroxidation inhibitors [18]. Based on these results and the traditional application thyme oil is frequently used in the treatment of respiratory diseases [28].

In Hungary, the cultivation of *T. vulgaris* is increasing and the scientific evaluation of the plant growing in private fields may provide interesting data for its further utilization. Therefore, the aim of this study was the antibacterial evaluation of *T. vulgaris* EO (TEO) collected during different flowering phenophases (at the beginning of the flowering period, in full bloom, at the end of the flowering period) against *H. influenzae*, *H. parainfluenzae*, and *P. aeruginosa* using thin layer chromatography-direct bioautography (TLC-DB) assay. The chemical composition of the TEOs was analyzed by gas chromatography-mass spectrometry (GC-MS) and gas chromatography-flame ionization detection (GC-FID). Moreover, anti-biofilm activity was tested against all Gram-negative pathogens mentioned above. The objective of the current study was to determine the optimal harvest time for *T. vulgaris* in connection with the highest antimicrobial and anti-biofilm activity of its EO content and to obtain of new scientific data on the changes of thyme volatile secondary metabolites in the course of its different flowering phenophases.

## Materials and methods

### Plant material and essential oil distillation

The plant samples were coded as follows: freshly distilled plant material collected at the beginning of the flowering period T1, at full bloom T2, at the end of the flowering period T3. For plants that were distilled after drying and collected at the beginning of the flowering period T4, at full bloom T5, at the end of the flowering period T6. In our previously published manuscript [29] EOs distilled from the fresh plant materials and collected at the beginning of flowering and at the end of flowering periods have already examined but in different in vitro assays with different aims. Therefore, here we would like to briefly describe the parameters. The collection of *T. vulgaris* L. was carried out at the beginning of the flowering period (23 May 2019), in full bloom (6 June 2019) and at the end of the flowering period (12 June 2019). Szigetvár city (Baranya county, Hungary, coordinates: (46◦02060.0000 N, 17◦47059.9900 E) was the harvesting field. The meteorological data are summarized in the Table 1. [30]. TEOs were obtained from the freshly collected and the dried plant materials by hydro distillation according to the Hungarian Pharmacopoeia 8th edition [31]. The drying process had been carried out at room temperature (23 °C) in the herb drying room of the Department of Pharmacognosy (University of Pécs, Pécs, Hungary) for one week. During the three flowering phenophases, the collected plant materials were halved, and one portion (fresh material) underwent the distillation process immediately, the other portion had been dried for one week (dried material).

The TEO content was measured with a volumetric method: 8450 µl was distilled from 1826 g fresh plant material collected at the beginning of flowering. 6350 µl and 3920 µl were isolated from 1464 g to 764 g fresh plant materials collected during in full bloom period and at the end of flowering, respectively. 7520 µl was distilled from 963 g dried plant material collected at the beginning of flowering. 6080 and 7550 µl were isolated from 964 to 1440 g dried plant materials collected in full bloom and at the end of flowering period, respectively.

The chemical composition of the TEOs was determined by gas chromatography-mass spectrometry (GC-MS) and gas chromatography-flame ionization detection (GC-FID) [31].

**Table 1:** Weather parameter records during specimen collection

	May	June
Maximum temperature	25.3 °C	34.7 °C
Minimum temperature	3.3 °C	12.9 °C
Rainy days	18 days	12 days
Rainfall	152 mm	95 mm
Sunny hours	202	336

### GC-MS and GC-FID

TEO samples were identified using a GCMS-QP2020 NX instrument (Shimadzu, Duisburg, Germany) equipped with an AOC-20i autosampler and a split-splitless injector. For chromatographic separation, a low-polarity capillary column, named SLB-5ms 30 m × 0.25 mm ID, 0.25 μm d_*f*_ (Merck KGaA, Darmstadt, Germany) was used. The injector temperature was set at 280 °C. The temperature program was as follows: 50 to 300 °C at 3.0 °C/min. Injection volume was 0.5 µL with a split ratio of 1:10. Helium was used as carrier gas at an initial inlet pressure of 26.7 kPa and an average linear velocity of 30 cm/s. The MS parameters included: mass range of 40–550 amu, ion source temperature of 220 °C, and interface temperature of 250 °C. GCMSsolution software (version 4.50 Shimadzu) was used for data collection and processing. Peak assignment was carried out using two different identification parameters: spectral similarity (over 85%) and LRI correspondence (± 5 units of tolerance) [31]. A homologous series C_7_–C_30_ saturated alkane standard mixture (Merck Life Science) in hexane (1000 mg/L) was used for LRI calculations. The FFNSC mass spectral library version 4.0 (Shimadzu) was used for the identification of peaks.

The quantification (area normalization) of compounds was carried out using a GC-2010 instrument (Shimadzu) equipped with an AOC-20i/s autosampler, a split-splitless injector, and an FID detector. The GC column, temperature program, and carrier gas were the same previously described for GC-MS analysis, except for the initial inlet pressure of carrier gas (99.5 kPa) at a linear velocity of 30 cm/s. The FID parameters included: temperature of 280 °C and sampling rate of 40 ms. Gas flows were as follows: 40 mL/min for hydrogen, 30 mL/min for the make-up gas (nitrogen), and 400 mL/min for air. Data were collected and processed using LabSolution software (version 5.92, Shimadzu).

### Antibacterial experiments

#### Cultivation of test bacteria

The antibacterial effect of TEOs was screened on *Haemophilus* spp., (*Haemophilus influenzae* DSM 4690; *H. parainfluenzae* DSM 8978) and *Pseudomonas aeruginosa* ATCC 27,853 as well. For TLC-DB assay, *Haemophilus* spp. were grown in 100 mL Brain Heart Infusion Broth (BHI) (Sigma Aldrich Ltd., Darmstadt, Germany) with 1 mL supplement B (Diagon Kft., Budapest, Hungary) and 15 µg/mL NAD solution (1 mg/mL). *P. aeruginosa* was grown in 100 mL BHI. Each bacterium was incubated in a shaker incubator (C25 Incubator Shaker, New Brunswick Scientific, Edison, New Jersey, USA) at 37ºC and at a speed of 60 rpm for 12 h [32]. The bacterial suspensions were diluted with fresh nutrient broth to an OD_600_ of 0.4, which corresponds to approximately 4 × 10^7^ colony-forming units (cfu) cfu/mL.

#### Thin layer chromatography-direct bioautography (TLC-DB)

The antibacterial effect of TEOs was investigated without TLC separation and after TLC separation [10, 33]. Based on this screening, the TEOs with the highest activity were selected for further assays, therefore, only TEOs distilled from fresh plant materials were involved into DB associated with TLC separation. Chromatography was performed on 10 × 10 cm silica gel 60 F_254_ aluminum sheet TLC plates (Merck, Darmstadt, Germany). TEOs were dissolved in absolute ethanol (stock solutions were 200 mg/mL), and 1.0 µL was applied to the TLC plate with Finnpipette pipettes (Merck, Darmstadt, Germany). Absolute ethanol was the negative control, and antibiotics were used as positive control. Ceftriaxone (Hospira, 250 mg powder, stock solution: 40 mg/mL) was applied against *Haemophilus* spp., and gentamicin (Sandoz, 40 mg/mL) was used against *P. aeruginosa*. From each antibiotic solution 1 µL was spotted on the TLC plate. During the TLC separation, the antibacterial activity of thymol as the main TEO component was also investigated by TLC-DB. Thymol (Spektrum-3D, Debrecen, Hungary) was dissolved in absolute ethanol (20 mg/mL). From the stock solution, 0.2 µL (0.004 mg) was applied to the plates. After sample application, the TLC plates were developed with toluene:ethyl acetate (95:5v/v) mobile phase [34]. Ascendant development chromatography was performed with a saturated twin trough chamber (Camag, Muttenz, Switzerland) and at room temperature (22 °C). After TLC separation, the absorbent layers were dried at 105 °C, for 5 min to remove the solvent completely. Ethanolic vanillin-sulfuric acid reagent was used to visualize the compounds of the TEOs. Separated compounds were detected based on Rf value and color of the standard. TLC plates were evaluated under UV light at 254 nm as well. It should be mentioned that the TLC plates for bioautography were not treated with ethanolic vanillin-sulfuric acid reagent, because this step interferes with the microbiological steps of TLC-DB.

After development, the layers were dipped into a 100 mL of bacterial suspension because of a homogenous distribution and adhesion of bacteria onto the surface of the layers. After immersion, the layers were placed into a low-wall horizontal chamber (chamber dimension: 20 × 14.5 × 5 cm) and incubated for 2 h at 37 °C. In order to visualize the antibacterial spots, TLC plates were immersed into the aqueous solution of 3-(4,5-dimethylthiazol-2-yl)-2,5-diphenyltetrazolium bromide (MTT, 0.05 g/75 mL) (Sigma Aldrich Ltd., Darmstadt, Germany), for 5 s, and then incubated at 37 °C for 12 h. On the TLC plate, metabolically active bacteria convert the MTT to formazan dye. White spots (as inhibition zones) against the bluish-violet background indicated the lack of dehydrogenase activity, due to the antibacterial activity of the tested TEOs or their main compound. The inhibitory zones (expressed in mm) of EOs without separation were measured with Motic Images Plus 2.0 program (ver. 2.0., Motic, Hong Kong, China).

### Preparing the stock solution containing thyme EO for broth microdilution and anti-biofilm assay

To dissolve the TEO in BHI, Tween40 (Sigma Aldricht Kft., Budapest, Hungary) was used as an emulsifier. 1% Tween40 was used for preparing the stock solution containing the TEO samples. In our experiments Tween40 did not show inhibitory effect as an emulsifier control [35].

### Broth microdilution test

During biofilm inhibition experiments minimum inhibitory concentration/2 (MIC/2) values of the TEOs distilled from fresh plant materials were used. The MICs were determined with broth microdilution test [23]. 96-well microtiter plates were used to perform this assay. From each bacterium solution (10^5^ cfu/mL) 100 µL was measured to the wells. In our preliminary experiment has shown that different concentrations (3, 3.5 and 5 mg/mL) of the TEOs’ stock solutions should be prepared to determine accurate MIC values. 3 mg/mL stock solutions were used from the ’full bloom’ and the ’end of flowering’ samples against *Haemophilus* spp, and ’the beginning of flowering’ TEO against *P. aeruginosa*. 3.5 mg/mL stock solutions were used from the ’end of flowering’ TEO sample against *P. aeruginosa*. 5 mg/mL stock solution were used in case of *Haemophilus* spp. from ’the beginning of flowering’ TEO sample. Each stock solution was prepared in BHI using 1% Tween40 as emulsifier and serial two-fold dilution was made up to (0.0468 mg/mL in case of 3 mg/mL stock solution, 0.0546 mg/mL in case of 3.5 stock solution and 0.0390 mg/mL in case of 5 mg/mL stock solution). After incubation (24 h, 37 °C) the absorbance was measured at 600 nm with plate reader (BMG Labtech, SPECTROstar Nano, Biotech Ltd., Szigetszentmiklós, Hungary). The negative control was the Tween40 solution, the positive control was the untreated bacterial suspension. The average of the six replicates was calculated and then the mean of the negative control was subtracted from the value obtained. Absorbance lower than 10% of the positive control samples, i.e. growth inhibition of 90% or more, was considered as the MIC value. During the assay, antibiotics controls were used as well. The antibiotic control was gentamicin (Gentamicin Sandoz, solution for injection, 80 mg/2 mL) against *P. aeruginosa*, ceftriaxone (Hospira, 250 mg powder, stock solution: 40 mg/mL) against *Haemophilus* spp.

### Anti-biofilm assay

The biofilm inhibitory activity was determined by crystal violet assay [36] as described in our previously published manuscript [10]. The TEOs were used in MIC/2 concentrations for the treatments. Tween40 (1%) was used as an emulsifier control.

In the biofilm inhibition assay, the activity of the TEOs was calculated and demonstrated in the term of inhibitory rate according to the following equation: Inhibitory rate = (1-S/C) * 100% (C and S were defined as the average absorbance of control and sample groups respectively [37].

### Scanning electron microscopy (SEM)

SEM was used to investigate the structural modifications of biofilms. Based on the result of anti-biofilm assay, TEO distilled from the fresh plant material and collected before flowering period was studied. For biofilm formation, 5 ml of BHI culture (10^8^ cfu/mL) of *P. aeruginosa*, *H. influenzae* or *H. parainfluenzae* were added into sterilized bottle. Sterile coverslips were placed in the bottle and served as the attaching surface for the cells. The plates were incubated for 4 h at 37 °C, then the planktonic cells and BHI were removed, and plates were rinsed with physiological saline. For the treatment of biofilms, 5 ml TEO in MIC/2 concentration was added. The untreated coverlips was used as control. After 24-h incubation time at 37 °C, the supernatant was removed, and the bottles were washed with physiological saline. The preparation of the samples for electron microscopy was performed with 2.5% glutaraldehyde for 2 h at room temperature to fix the biofilms formation. In order to dehydrate the biofilms, different ethanol concentrations (50, 70, 80, 90, 95, 98%) were used at room temperature for 30 min. The last step of dehydration was the t-butyl alcohol: absolute ethanol solution in 1:2, 1:1 and 2:1 ratios. For each ratio, the dehydration lasted for 1 h at room temperature, then dehydrated with absolute t-butyl alcohol for 2 h at room temperature. The samples were stored at 4 °C for 1 h and freeze-dried overnight. The samples were coated with a gold membrane and observed with JEOL JSM IT500-HR scanning electron microscope (Jeol Ltd., Tokio, Japan) [38].

### Statistical analyses

Statistical analyses were performed in R, version 4.0.2 (R Development Core Team 2020). The measured inhibition zones were analyzed with linear model [39] using the function lm. In our model the explanatory variables (bacteria species, phenophase and status of thyme), treated as fixed factors. No data transformation was made, due to the normal distribution of the data. Checking the need of transformation was based on graphical evaluation according to [40]. Statistical hypothesis testing was performed with ANOVA test. For pair-wise comparisons, Tukey post-hoc tests were conducted in with multcomp-package [41] to compare the difference among all experimental set-ups.

## Results

### Plant distillation

Table 2 contains the yields of the essential oils distilled from our thyme samples. Based on our results, it can be concluded that the yield of sample T4 (TEO distilled from dried plant material and collected at the beginning of flowering period) was the highest.

**Table 2:** Essential oil yields detected during the distillation. The TEO yields were expressed in mL/100 g

Abbreviations of the samples and their essential oil yields (in mL/100 g)		
T1	TEO distilled from fresh plant material and collected at the beginning of flowering period	0.46
T2	TEO distilled from fresh plant material and collected during full bloom	0.43
T3	TEO distilled from fresh plant material and collected at the end of flowering period	0.51
T4	TEO distilled from dried plant material and collected at the beginning of flowering period	0.78
T5	TEO distilled from dried plant material and collected during full bloom	0.63
T6	TEO distilled from dried plant material and collected at the end of flowering period	0.52

### Chemical composition of essential oils

In our previous manuscript [29], the chemical composition of TEOs isolated from fresh plant materials and collected at the beginning of flowering (T1) and end of flowering periods (T3) have already been published. Therefore, here the composition of TEOs isolated from fresh plant materials and collected in full bloom, isolated from dried materials and collected at the three investigated phenophases are described. Identified compounds and their percentage value of the TEOs are shown in Table 3. The main component of each thyme oil was thymol regardless of the collection time as well as the moisture content of the plant. The highest thymol content (62.4%) was detected during full bloom period and from dried plant material. In the fresh plant material collected in full bloom thymol showed 57.1% value. The lower thymol content was measured in plant material collected at the end of flowering period (fresh sample: 54.2%, dried sample: 52.3%). Besides thymol, *p*-cymene and *γ*-terpinene were represented in significant amount. The highest *p*-cymene value (22.7%) was detected in TEO distilled from dried plant material and collected at the end of flowering period, but this component was significant in the fresh plant material collected in the same phenophase (20.6%). *γ*-Terpinene content was the highest in the TEO isolated from fresh material and collected at the beginning of flowering period. Other minor components include myrcene (0.7-1.4%), *α*-terpinene (0.7-1.4%), linalool (1.4-2.1%), carvacrol (2.3-3.4%) and (*E*)-caryophyllene (1.5-2.5%).

**Table 3:** List of terpene and terpenoid compounds detected in TEOs prepared from fresh and dried plant materials and collected at different flowering phenophases. Quantities are expressed as relative abundance (area %)

Compounds	MS Sim (%)	LRI _Exp_	LRI _Ref_	T2	T4	T5	T6
Tricyclene	94	922	923	0.02	0.01	0.01	0.02
*α*-Thujene	98	925	927	1.09	0.46	0.21	0.37
*α*-Pinene	97	933	933	0.63	0.50	0.37	0.55
Camphene	97	949	953	0.39	0.33	0.25	0.42
Sabinene	95	972	972	0.02	0.01	0.01	0.01
Pent-4-enyl propanoate	94	974	974	0.01	0.04	0.01	0.01
*β*-Pinene	92	977	978	0.20	0.15	0.12	0.15
Vinyl amyl carbinol	95	979	978	0.34	0.36	0.34	0.51
Octan-3-one	93	984	986	0.03	0.04	0.02	0.03
Myrcene	96	988	991	1.34	1.17	0.71	0.84
Octan-3-ol	96	997	999	0.03	0.02	0.02	0.03
*α*-Phellandrene	96	1006	1007	0.12	0.13	0.08	0.08
*δ*-3-Carene	96	1009	1009	0.08	0.07	0.05	0.06
*α*-Terpinene	98	1017	1018	1.09	1.15	0.74	0.79
*p*-Cymene	96	1025	1025	17.44	12.46	15.02	22.78
Limonene	96	1029	1030	0.30	0.27	0.22	0.31
*β*-Phellandrene	94	1030	1031	0.10	0.08	0.08	0.11
Eucalyptol	97	1032	1032	0.70	0.50	0.67	0.90
(*Z*)-, β-Ocimene	90	1034	1035	0.01	0.01	0.00	0.01
(*E*)-, β-Ocimene	95	1045	1046	0.02	0.04	0.02	0.02
*γ*-Terpinene	95	1058	1058	7.38	13.67	5.06	5.49
3-Methylbut-2-enyl butanoate	90	1063	1068	0.06	0.06	0.05	0.05
(*Z*)-Sabinene hydrate	93	1070	1069	0.33	0.21	0.31	0.21
Terpinolene	96	1086	1086	0.09	0.10	0.10	0.11
*p*-Cymenene	94	1091	1093	0.02	0.02	0.03	0.05
Linalool	97	1099	1101	1.56	1.69	1.67	2.17
(*E*)-Sabinene hydrate	94	1102	1099	0.13	0.10	0.14	0.11
3-Methylbut-3-enyl 3-methylbutanoate	90	1110	1114	*tr*	*tr*	0.01	0.02
(*Z*)-, *p*-Menth-2-en-1-ol	96	1126	1124	0.03	0.03	0.04	0.05
Camphor	97	1149	1149	0.25	0.26	0.29	0.33
Borneol	98	1173	1173	0.44	0.49	0.51	0.85
Terpinen-4-ol	92	1182	1184	0.75	0.67	0.67	0.87
Hex-(3*Z*)-enyl-Butyrate	92	1184	1187	0.03	0.03	0.04	0.04
*p*-Cymen-8-ol	93	1189	1189	0.04	0.04	0.05	0.08
*α*-Terpineol	97	1197	1195	0.15	0.13	0.16	0.21
(*Z*)-, Dihydro-carvone	94	1200	1198	0.05	0.05	0.06	0.07
*n*-Decanal	95	1206	1208	0.01	0.01	*nd*	*nd*
Thymol methyl ether	94	1230	1229	0.50	0.57	0.43	0.53
Carvacryl methyl ether	96	1239	1239	0.36	0.35	0.32	0.32
Neral	96	1242	1238	0.01	0.01	0.01	0.01
Carvone	95	1249	1246	0.02	0.02	0.01	0.02
Geranial	97	1274	1268	0.02	0.02	0.01	0.02
Thymol	94	1294	1293	57.10	56.39	62.46	52.33
Carvacrol	94	1302	1300	2.92	2.98	3.48	3.11
Thymol acetate	93	1345	1348	0.02	0.03	0.01	*nd*
Eugenol	95	1354	1357	0.09	0.02	0.01	0.05
*α*-Ylangene	92	1371	1371	*nd*	0.02	0.02	0.02
Isobornyl propionate *	93	1376	1377	0.04	0.07	0.09	0.10
*α*-Copaene *	88	1377	1375				
*β*-Bourbonene	95	1385	1382	0.03	0.03	0.04	0.06
(*Z*)-Jasmone	93	1394	1394	0.01	*tr*	0.01	*tr*
(*E*)-Caryophyllene	97	1421	1424	2.05	2.15	2.50	2.44
*β*-Copaene	94	1431	1433	0.02	0.03	0.03	0.03
Aromadendrene	94	1441	1438	*nd*	0.04	0.01	0.01
*α*-Humulene	97	1457	1454	0.06	0.07	0.07	0.07
(*Z*)-Muurola-4(14),5-diene	94	1464	1466	0.01	0.01	0.01	*tr*
Geranyl propanoate	97	1468	1471	0.05	0.08	0.07	0.06
Cadina-1(6),4-diene	91	1473	1474	*nd*	0.01	0.02	*tr*
*γ*-Muurolene	92	1476	1478	0.07	0.14	0.14	0.12
*α-*Amorphene	90	1481	1482	0.01	0.02	0.02	0.02
Germacrene D	95	1482	1480	0.07	*nd*	*nd*	*nd*
*β*-Selinen	94	1491	1492	0.01	0.01	0.01	0.01
*γ*-Amorphene	87	1494	1490	0.02	0.06	0.05	0.04
*α*-Selinene	89	1497	1501	0.01	0.01	0.02	0.02
*α*-Muurolene	93	1500	1497	0.03	0.05	0.06	0.05
*δ*-Amorphene	89	1505	1506	*nd*	0.02	0.01	*tr*
*γ*-Cadinene	95	1515	1512	0.12	0.16	0.24	0.17
*δ*-Cadinene	94	1520	1518	0.13	0.26	0.27	0.22
(*E*)-Calamenene	90	1522	1527	0.04	0.03	0.07	0.06
(*E*)-Cadina-1,4-diene	93	1534	1536	0.01	0.02	0.02	0.02
α-Cadinene	95	1539	1538	0.01	0.02	0.03	0.02
α-Calacorene	91	1543	1544	*nd*	*tr*	0.01	0.01
Geranyl butyrate	97	1554	1559	0.02	0.02	0.03	0.03
Caryophyllene oxide	93	1585	1587	0.37	0.52	0.70	0.70
Humulene epoxide II	89	1613	1613	0.01	0.01	0.02	0.01
1-,10-di-*epi*-Cubenol	89	1618	1614	0.02	0.01	0.03	0.01
*epi*-γ-Eudesmol	95	1626	1624	0.04	0.03	0.03	0.03
*α*-Cadinol	94	1645	1641	0.17	0.07	0.17	0.05
Cadin-4-en-10-ol	95	1658	1659	0.03	0.02	0.02	0.01
Not identified				0.22	0.26	0.30	0.51
Total				100.00	100.00	100.00	100.00

**Abbreviations**: MS Sim (%), mass spectral similarity; LRI _Exp_, experimental linear retention index; LRI _Ref_, reference linear retention index; *nd*, not detected; *tr*, trace level; * compounds coeluted on SLB-5-ms GC column. T2: TEO distilled from fresh plant material and collected in full bloom, T4: TEO distilled from dried plant material and collected at the beginning of flowering period, T5: TEO distilled from dried plant material and collected in full bloom, T6: TEO distilled from dried plant material and collected at the end of flowering period.

### Antibacterial experiments

#### TLC-DB

**Fig. 1:**
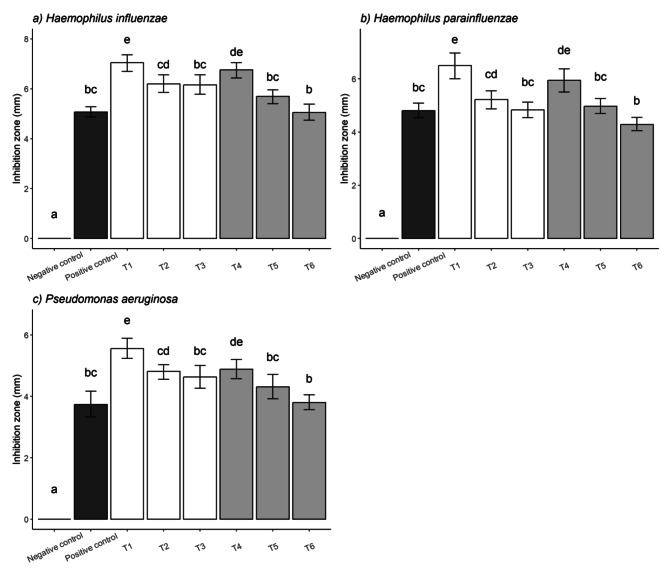
Antibacterial activity of TEOs with TLC-DB (without TLC separation)

In the TLC-DB method, the activity of the TEOs without and with chromatographic separation was tested against *Haemophilus* spp. and *P. aeruginosa*. In the case of activity of the EOs without separation (Fig. 1), the activity of the “total” extract (distilled TEO) was examined. The diameter of the inhibition zones was expressed in mm. From the stock solution of EOs 1 µL was applied (equivalent to 0.2 mg undiluted EO) on the TLC plate. The *Haemophilus* spp. was more sensitive to the EOs than *P. aeruginosa.* Absolute ethanol as the negative control did not inhibit the growth of both bacteria. The 1 µL solution of the antibiotics sample (ceftriaxone against *Haemophilus* spp., gentamicin against *P. aeruginosa*) was effective against the tested bacteria. Generally, thyme oils from the fresh plant materials showed higher antibacterial activity than the thyme oils from the dried plant materials. The TEO distilled from fresh plant and collected at the beginning of flowering period (T1) presented the highest activity in case of *Haemophilus influenzae* (7.04 mm), *H. parainfluenzae* (6.5 mm), and *P. aeruginosa* (5.5 mm) as well. However, the TEOs distilled from the fresh plant material and collected during full bloom (T2) and at the end of flowering period (T3) had antibacterial effect as well (in full bloom: *H. influenzae* − 6.3 mm, *H. parainfluenzae* − 5.2 mm, *P. aeruginosa* − 4.9 mm; at the end of flowering: *H. influenzae* − 6.15 mm, *H. parainfluenzae* − 4.8 mm, *P. aeruginosa* − 4.5 mm). The smallest zones of inhibition were detected with TEO distilled from dried material and collected at the end of flowering period (T6) (*H. influenzae* − 5 mm, *H. parainfluenzae* − 4.8 mm, *P. aeruginosa* − 3.7 mm). Overall, the TEOs distilled from the fresh plant samples proved to be more effective than the samples distilled from the dried plant materials. Furthermore, regarding the flowering phenophase, the EO of the plant material collected at the beginning of flowering period was the most effective. The antibiotic controls were more efficient than the TEO samples with the concentrations used in this study.

The diameter of the inhibition zones was expressed in mm. Negative control: absolute ethanol; positive control: ceftriaxone against *Haemophilus* spp., gentamicin against *P. aeruginosa* (equivalent to 0.04 mg antibiotic); 1 µL of EO samples (equivalent to 0.2 mg undiluted EO) was applied. T1: TEO distilled from fresh plant material and collected at the beginning of flowering period, T2: TEO distilled from fresh plant material and collected in full bloom, T3: TEO distilled from fresh plant material and collected at the end of floweing period, T4: TEO distilled from dried plant material and collected at the beginning of flowering period, T5: TEO distilled from dried plant material and collected in full bloom, T6: TEO distilled from dried plant material collected at the end of flowering period. Error bars represent S.E.M. Lowercase letters (a–e) show pairwise comparison based on Tukey post-hoc test, *p* < 0.05.

**Fig. 2:**
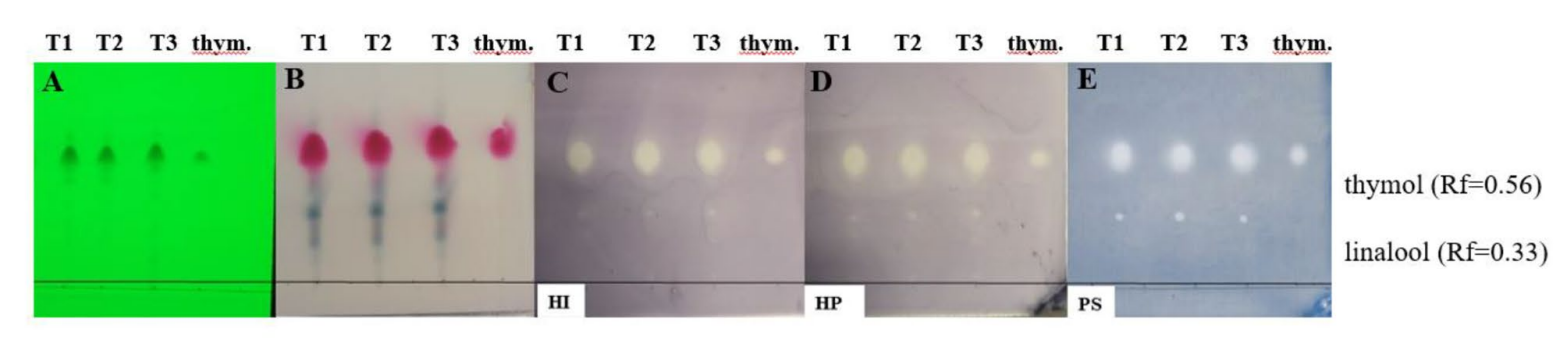
Antibacterial components in the TEOs distilled from fresh plant material after TLC-DB associated with chromatographic separation. Mobile phase: toluene-ethyl acetate 95:5 (v/v); 1 µL indicated the applied volumes of the EOs and 0.2 µL for the standard. (**A**) TLC plate under UV 254 nm, (**B**) TLC plate after treatment with vanillin-sulfuric acid reagent and documented in visible light, (**C**) TLC-DB assay: Bioautograms using *H. influenzae*, (**D**) TLC-DB assay: Bioautograms using *H. parainfluenzae*, (**E**) TLC-DB assay: Bioautograms using *P. aeruginosa* (bright zones indicate antibacterial effects); T1-TEO distilled from fresh plant material and collected at the beginning of flowering period (200 mg/mL), T2-TEO distilled from fresh plant material and collected in full bloom (200 mg/mL), T3-TEO distilled from fresh plant material and collected at the end of flowering period (200 mg/mL), thym.-thymol standard (20 mg/mL)

TLC-DB is a suitable assay for the examination of the antibacterial activity of separated compounds in EO samples. Based on the result of TLC-DB without separation, we included only the TEOs distilled from fresh plant material into TLC-DB associated with chromatographic separation. In the TEOs, thymol component (at Rf = 0.56), as well as its standard showed activity against all of the tested bacteria. At Rf = 0.33, linalool was identified as an active compound according to the GC-MS result and Wagner and Bladt [42] (Fig. 2).

### Biofilm inhibition experiments

#### Broth microdilution assay

Based on the TLC-DB results, the TEO samples distilled from the fresh plant materials were used in this assay and in the biofilm inhibition experiment. The most sensitive strains to the EO treatment were *H. influenzae* and *H. parainfluenzae*, their growth was inhibited by 0.156–0.187 mg/mL MIC value of EOs (Table 4.). In case of *P. aeruginosa*, higher MIC values, 1.5–1.75 mg/mL were measured. The most effective TEO was distilled from the plant material collected at the beginning of flowering period. In the biofilm inhibition assay MIC/2 values were used [38].

**Table 4:** The MIC values of thyme EOs (mg/mL), and antibiotics (µg/mL) on respiratory bacteria

	EOs	*H. influenzae*	*H. parainfluenzae*	*P. aeruginosa*
	at the beginning of flowering	0.156	0.156	1.500
**MIC value**	in full bloom	0.187	0.187	1.750
	at the end of flowering	0.187	0.187	1.750
	gentamicin	-	-	6.3
	ceftriaxone	1.5	1.5	-

#### Anti-biofilm assay

**Fig. 3:**
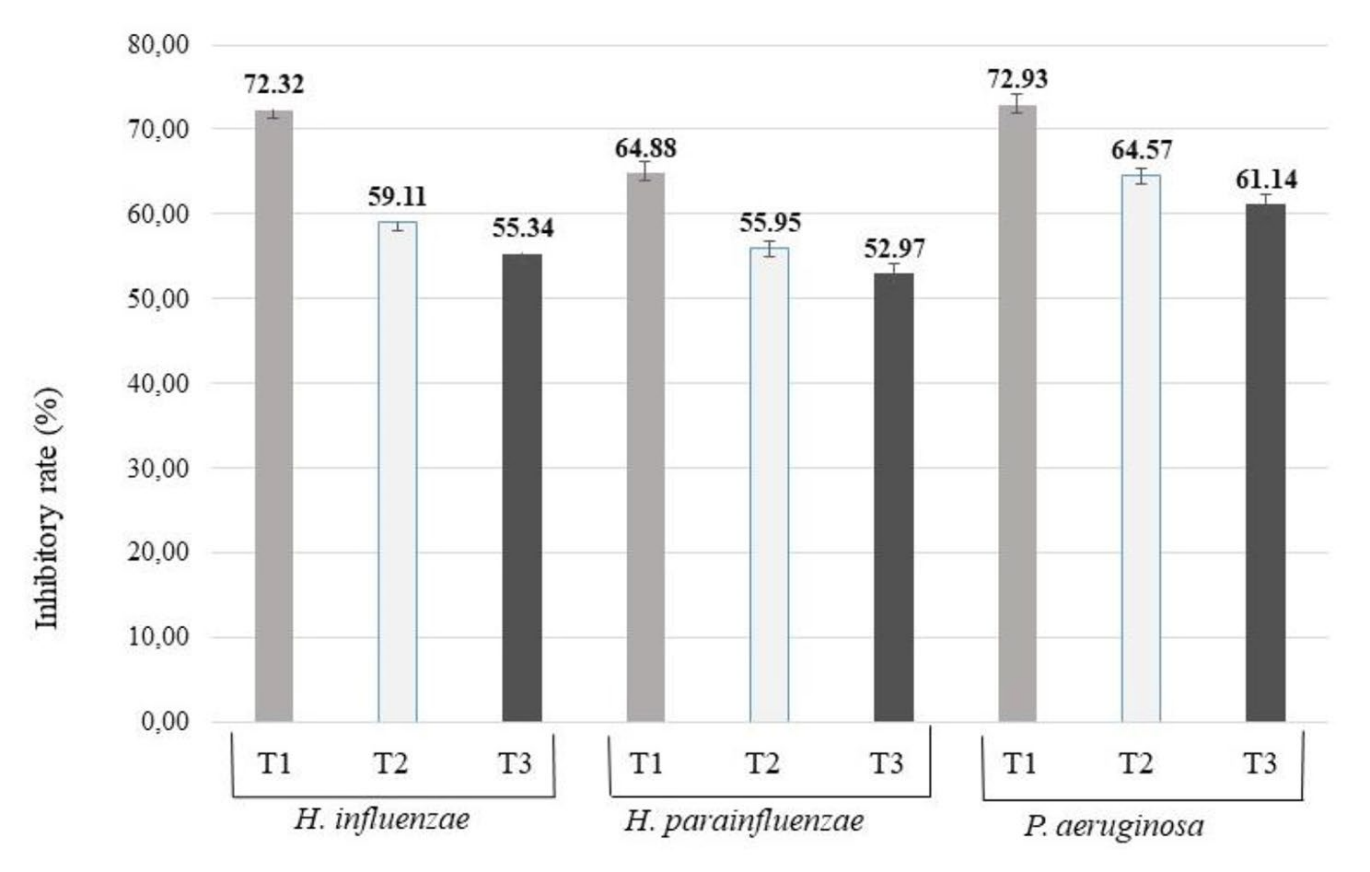
Biofilm inhibition activity of thyme EOs distilled from fresh plant material and collected at different flowering phenophases

Our results showed that each TEO sample could inhibit the biofilm formation (Fig. 3). TEO distilled from plant material and collected at the beginning of flowering period was the most effective against all of the tested bacteria used in our study. This EO showed the highest inhibitory rate, 72.93%, against *P. aeruginosa*. In case of *H. influenzae* and *H. parainfluenzae*, 72.32% and 64.88% inhibitory rates were calculated after the treatment with TEO distilled from plant material and collected at the beginning of flowering period, respectively. The results from the biofilm degradation assay support the TLC-DB results. In both experiments, the TEO distilled from plant material and collected at the beginning of flowering period was the most effective against all of the tested bacteria.

T1: TEO distilled from fresh plant material and collected at the beginning of flowering period, T2: TEO distilled from fresh plant material and collected in full bloom, T3: TEO distilled from fresh plant material and collected at the end of flowering period.

The inhibitory rate was calculated based on the following equation: Inhibitory rate = (1 - S/C) x 100% (C and S were defined as the average absorbance of control and sample groups, respectively).

#### Detection of biofilm structure with SEM

Based on the results of TLC-DB and biofilm inhibition assays, the effect of TEO distilled from fresh plant material and collected at the beginning of flowering period (T1) was investigated with SEM.

**Fig. 4:**
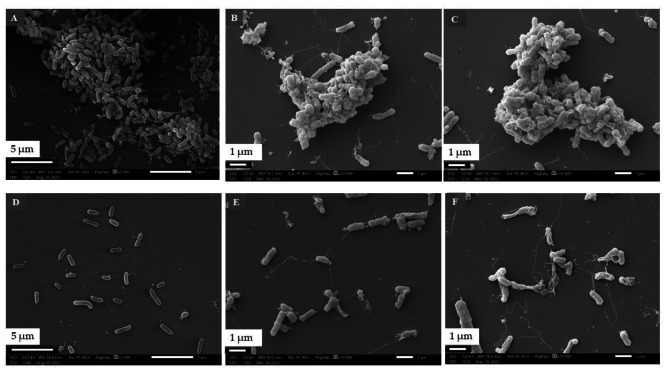
Scanning electron microscopic images of *P. aeruginosa* (**A, D**), *H. influenzae* (**B, E**) and *H. parainfluenzae* (**C, F**) biofilms

The images of the control samples (without EO treatment) captured the characteristic morphological elements of a mature, three-dimensional biofilm (Fig. 4A, B, C). The EO treatment resulted that the bacterial cells attached to the surface, but they did not form biofilm-specific structures (Fig. 4. D, E, F).

A-C: Control samples of bacterial strains: A - *P. aeruginosa*, B - *H. influenzae*; C - *H. parainfluenzae.* D-F: Treated samples with T1 TEO (in MIC/2): D - *P. aeruginosa*, E - *H. influenzae*; F - *H. parainfluenzae*.

## Discussion

The constantly growing antibiotic resistance among pathogenic microorganisms has driven researchers to search novel antimicrobials. This fact may support that the antimicrobial potentials of EOs and their respective constituents have been widely investigated. EOs with their complex chemical composition offer promising solution to treat the antibiotic resistance problem. Several publications have already described their antimicrobial activity and mode of action [43–46].

The cultivation of medicinal plants having EO content is increasing in several countries. The scientific evaluation of these cultivation fields would be highly welcomed and provides valuable data when these medicinal plants have to be harvested and provide biologically active volatile oils. Therefore, in our study we focused on the antibacterial activity of thyme EO cultivated in Hungary, Baranya County. Our research team evaluated this field for the first time.

TLC-DB revealed that TEOs were effective against all tested bacteria. The *Haemophilus* spp. was more sensitive to the TEOs than *P. aeruginosa*. Overall, the TEOs distilled from the fresh plant samples proved to be more effective than the samples distilled from the dried plant materials. Furthermore, regarding the flowering phenophase, the TEO of the plant material collected at the beginning of flowering period was the most effective. The antibiotic controls were more efficient than the TEOs samples with the concentrations used in this study.

Galasso et al. described that the chemical composition and biological effect of *T. longicaulis* oil is influenced by the season of harvest [47]. Our results also show that the composition of *T. vulgaris* oil changes during the flowering phenophases. The main component of TEOs was thymol, which is not solely responsible for the biological effect. The proportion of active components varied similarly in the case of dried and freshly distilled samples [47]. The proportion of *p-*cymene in the EOs was the highest at the end of the flowering period in both cases. The highest thymol content was measured during the full flowering period. Nevertheless, the *γ*-terpinene content at the beginning of the flowering period was almost three times higher than in the other phenophases. However, the samples that were collected at the beginning of the flowering phase had the highest antibacterial effect. This may be due to the addictive and synergistic effect of the components of EOs [48–53]. The antibacterial effect of thymol and *p*-cymene was investigated by Gömöri et al. individually and in combination. Thymol alone showed a higher antibacterial effect than *p*-cymene alone or the combination of the two components in any ratio. However, EO with reduced thymol and increased *p*-cymene and *γ* -terpinene content showed higher antimicrobial activity in some cases [47]. This correlated with our results. In our case, the ratio of *γ*-terpinene was significantly increased in plant materials collected at the beginning of flowering. However, the thymol content was slightly lower than in the full bloom period. The high proportion of *γ-*terpinene at the beginning of flowering is logical, as it is the initial molecule for the biosynthesis of thymol and carvacrol. Linalool was also identified as an active compound by TLC-DB associated with chromatographic separation. The article ‘Ph.Eur. 01/2012:1374 Thyme oil’ specifies the chemical composition of thyme oil, particularly with respect to its main components. According to the pharmacopoeial specification, the percentage of *γ*-terpinene in thyme oil should not exceed 12%. Only the plant collected at the beginning of flowering had a higher *γ*-terpinene content (13.67%) which showed the best antibacterial effect. In fact, almost all the samples have a high thymol content, because the upper limit in the article is 55%. The only sample that meets the chemical composition requirements of the pharmacopoeia is the oil from the plant sample collected at the end of flowering period. Although, in the TLC-DB assay without separation, samples collected at the end of the flowering period showed the lowest antibacterial effect.

Several studies have compared the antimicrobial activity of different chemotypes of *T. vulgaris* against various pathogens, including *P. aeruginosa* and *Haemophilus* spp [54–56]. Two chemotypes, *T. vulgaris* ct. thymol and *T. vulgaris* ct. carvacrol, have shown the strongest antibacterial effects against these bacteria. Geraniol and *trans*-thujan-4-ol/terpinen-4-ol chemotypes were less effective against *P. aeruginosa* [57]. Our results support previous findings indicating that thyme essential oil of the thymol chemotype exhibits efficacy against the tested bacteria.

The results from the biofilm inhibition assay support the TLC-DB results. In both experiments, the TEO distilled from fresh plant material and collected at the beginning of flowering period was the most effective against all of the tested bacteria. Sessile biofilm bacteria are phenotypically different from planktonic bacteria. Planktonic cells are released by the biofilm after the maturation phase. While the antibiotics destroy the planktonic cells, the bacteria within the biofilm are able to survive and cause chronic and antibiotic-resistant infections [57–61]. Ability of *Haemophilus* spp. and *P.aeruginosa* forming a biofilm plays an important role, because the bacterial cells are located very close to each other, which accelerates the transmission of genes carrying resistance by horizontal gene transfer [61–66]. TEO reduces bacterial cell membrane hydrophobicity and proteinase and hemolysin production, thereby preventing bacterial biofilm formation [66–69]. Kerekes et al. similar to our results, by SEM images displayed with a confocal laser scanning electron microscope also showed that the three-dimensional structure of the mature biofilm was disintegrated after TEOs treatment [35].

Collectively, our findings suggest that thyme oil distilled from fresh plant material and collected at the beginning of flowering period exerted the highest antibacterial and anti-biofilm activity against *Haemophilus influenzae*, *H. parainfluenzae* and *Pseudomonas aeruginosa*. We have shown that the structure of the bacterial cell membrane was damaged, which resulted in the breakdown of the structure of the bacterial biofilm.

## Conclusion

In conclusion, the different flowering periods of *T. vulgaris* influence the antibacterial and anti-biofilm activity of its EO, therefore, the collection time has to be taken into consideration and not only the full bloom, but the beginning of flowering period may provide biologically active thyme EO. The TEO isolated from fresh plant material was more effective in the microbiological assays then the TEO isolated from dried plant material. We also proved that the thymol chemotype of *T. vulgaris* essential oil is effective antibacterial and anti-biofilm oil in in vitro experiments used in this study.

## Data Availability

The datasets used for the current study are available from the corresponding author upon request. Furthermore, all the necessary data used to support the results of this study are included in the manuscript.

## List of Abbreviations

BHI: Brain heart infusion broth
cfu: Colony-forming unit
EO: Essential oil
GC-FID: Gas chromatography-flame ionization detection
GC-MS: Gas chromatography-mass spectrometry
*Haemophilus* spp.: *Haemophilus influenzae*; *Haemophilus parainfluenzae*
MIC: Minimum inhibitory concentration
MRSA: Methicillin-resistant *Staphylococcus aureus*
MTT: 3-(4,5-dimethylthiazol-2-yl)-2,5-diphenyltetrazolium bromide
NAD: Nicotinamide adenine dinucleotide
*P. aeruginosa*: *Pseudomonas aeruginosa*
SEM: Scanning electron microscopy
TEO: Thyme essential oil
*T. vulgaris*: *Thymus vulgaris* L
TLC-DB: Thin layer chromatography-direct bioautography
